# Micro-and mesoscale aspects of neurodegeneration in engineered human neural networks carrying the LRRK2 G2019S mutation

**DOI:** 10.3389/fncel.2024.1366098

**Published:** 2024-04-05

**Authors:** Vibeke Devold Valderhaug, Ola Huse Ramstad, Rosanne van de Wijdeven, Kristine Heiney, Stefano Nichele, Axel Sandvig, Ioanna Sandvig

**Affiliations:** ^1^Department of Research and Innovation, Møre and Romsdal Hospital Trust, Ålesund, Norway; ^2^Department of Neuromedicine and Movement Science, Faculty of Medicine, Norwegian University of Science and Technology (NTNU), Trondheim, Norway; ^3^Department of Clinical and Molecular Medicine, Faculty of Medicine and Health Sciences, NTNU, Trondheim, Norway; ^4^Department of Computer Science, Faculty of Technology, Art and Design, Oslo Metropolitan University (OsloMet), Oslo, Norway; ^5^Department of Computer Science, Faculty of Information Technology and Electrical Engineering, NTNU, Trondheim, Norway; ^6^Department of Computer Science and Communication, Østfold University College, Halden, Norway; ^7^Department of Clinical Neuroscience, Division of Neuro, Head and Neck, Umeå University Hospital, Umeå, Sweden; ^8^Department of Community Medicine and Rehabilitation, Umeå University, Umeå, Sweden; ^9^Department of Neurology and Clinical Neurophysiology, St Olav’s Hospital, Trondheim, Norway

**Keywords:** structure–function, LRRK2 G2019S mutation, human neural networks, mitochondrial dynamics, Parkinsons disease (PD), neurodegenerative disease model

## Abstract

Mutations in the leucine-rich repeat kinase 2 (LRRK2) gene have been widely linked to Parkinson’s disease, where the G2019S variant has been shown to contribute uniquely to both familial and sporadic forms of the disease. LRRK2-related mutations have been extensively studied, yet the wide variety of cellular and network events related to these mutations remain poorly understood. The advancement and availability of tools for neural engineering now enable modeling of selected pathological aspects of neurodegenerative disease in human neural networks *in vitro*. Our study revealed distinct pathology associated dynamics in engineered human cortical neural networks carrying the LRRK2 G2019S mutation compared to healthy isogenic control neural networks. The neurons carrying the LRRK2 G2019S mutation self-organized into networks with aberrant morphology and mitochondrial dynamics, affecting emerging structure–function relationships both at the micro-and mesoscale. Taken together, the findings of our study points toward an overall heightened metabolic demand in networks carrying the LRRK2 G2019S mutation, as well as a resilience to change in response to perturbation, compared to healthy isogenic controls.

## Graphical abstract

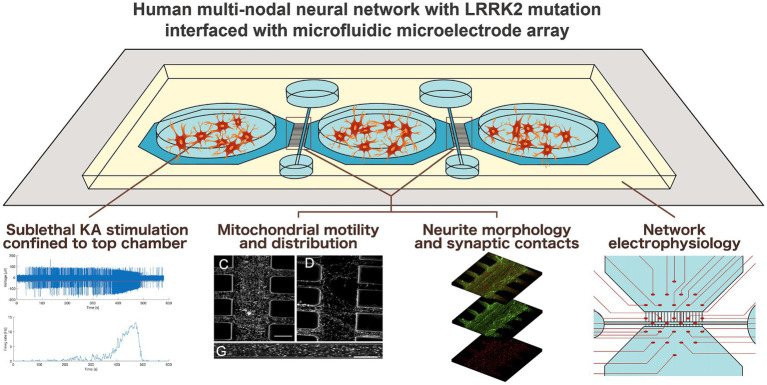

## Introduction

Advances in neural engineering now enable modeling of selected pathological aspects of neurodegenerative disease in human neural networks *in vitro* (Levi et al., 2021; Valderhaug et al., 2021; Estévez-Priego et al., 2023). Of particular interest is how neurodegenerative pathology may manifest and affect network behavior at the microscale and mesoscale, i.e., before it progresses to affect the entire system. The relevant questions are highly challenging or per definition not feasible to address using *in vivo* models, thus advanced neural engineering models represent a powerful alternative or complementary approach. A fundamental property of such models is that neurons *in vitro* maintain intrinsic behavior of neurons in the brain by self-organizing over time into networks of increasing structural and functional complexity (Innocenti and Price, 2005; Chiappalone et al., 2006; Giugliano et al., 2006). By combining custom-designed microfluidics devices (MFDs) with microelectrode arrays (MEAs), it is possible to structure multi-nodal human neural networks with controllable connectivity and study both intrinsic network behavior as well as responses to inherent or selectively induced perturbations (van de Wijdeven et al., 2018, 2019; Valderhaug et al., 2019, 2021; Fiskum et al., 2021, 2024; Levi et al., 2021; Heiney et al., 2022; Winter-Hjelm et al., 2023; Weir et al., 2023a,b).

Mutations in the leucine-rich repeat kinase 2 (LRRK2) gene are linked to both late-onset familial and sporadic forms of Parkinson’s disease (PD) (Healy et al., 2008). Although rare, LRRK2 gene mutations have been termed a potential “Rosetta stone” of parkinsonian disorders as all of the major pathologies related to parkinsonism have been observed, in addition to there being end-stage variability, within families carrying the same pathogenic variant (Trinh et al., 2006). The LRRK2 gene is expressed both in the brain and in other tissues throughout the body and is translated into the LRRK2 protein, which has enzymatic kinase activity involved in a range of cellular processes (Trinh et al., 2006). In a recent proteome wide association study of LRRK2 variants from the Parkinsons progression markers initiative cohort (PPMI) (Marek et al., 2018) the LRRK2 gene was directly linked to the regulation of several PD-associated proteins, many of which were subsequently identified as enriched in specific endolysosomal pathways, in microglial cells, and in immune response (Phillips et al., 2023). Among the LRRK2 mutations, the particular G2019S variant represents the most commonly identified cause of late-onset PD, and has been shown to contribute uniquely to both familial and sporadic forms of the disease (Di Fonzo et al., 2005; Gilks et al., 2005; Kachergus et al., 2005; Lesage et al., 2005; Nichols et al., 2005; Ozelius et al., 2006; Trinh et al., 2006; Healy et al., 2008; Moore, 2008; Bouhouche et al., 2017). Moreover, this mutation has been shown to result in differential regulation of a variety of cellular pathways highly relatable to several aspects of PD pathology, among which are axonal guidance, mitochondrial function and calcium homeostasis, cytoskeletal transport, lysosomal function, cell growth, differentiation, and synaptic function (Habig et al., 2008, 2013; Ludtmann et al., 2019; Aleknonytė-Resch et al., 2023; Ohtonen et al., 2023).

We hypothesized that neural networks carrying the Parkinson’s related LRRK2 G2019S mutation would display inherently different micro-and mesoscale behaviors compared to isogenic healthy controls, both during development and in response to induced perturbation. To address this hypothesis, we engineered multi-nodal neural networks from iPSC-derived human neural stem cells with and without the LRRK2 G2019S mutation using a custom-designed MFD (van de Wijdeven et al., 2018, 2019), allowing for investigation of structure–function relationships. Specific microtopographies embedded in the MFD, including axon tunnels and synaptic compartments, allowed for the selective manipulation and targeted investigation of neurites and their organelles, as well as their synaptic elements, i.e., network sites particularly related to early manifestation of neurodegenerative disease pathology. Furthermore, the electrophysiological profile of the multi-nodal neural networks from each group was evaluated in parallel assays by combining the MFDs with an MEA interface.

Monitoring the early network structure and function revealed distinct differences both at the micro-and mesoscale between the LRRK2 G2019S and healthy control neural networks. Specifically, we found that the LRRK2 G2019S neurons self-organized into networks with heightened metabolic demand – exhibiting aberrant morphology and containing neuritic mitochondria moving at higher speeds, compared to the healthy isogenic controls. Inducing a transient, confined overexcitation in targeted nodes of the neural networks further revealed differences in mitochondrial motility and neuritic remodeling between the groups. Together with the indicated difference in total network correlation profiles 24 h post perturbation, these dissimilarities point toward a resilience to change in the LRRK2 G2019S neural networks.

## Materials and methods

### Structuring multi-nodal cortical neural networks using MFDs with directional connectivity

Human induced pluripotent stem cell (iPSC)-derived H9N neural stem cells (NSCs) homozygously carrying the LRRK2 G2019S (GGC > AGC) mutation (ax0310, Axol Bioscience, Cambridge, United Kingdom) and healthy isogenic control iPSC-derived H9N NSCs (ax0019, Axol Bioscience, UK) were cultured and expanded on 0.01% poly-L-ornithine (PLO) (P4967, Sigma-Aldrich, US) and L-15 laminin (L15 medium, L5521, Sigma-Aldrich) containing 1:60 natural mouse laminin (23017015, Thermo Fisher Scientific) and 1:41 sodium bicarbonate) coated culture vessels in neural expansion medium (ax0030, Axol Bioscience, UK) supplemented with human FGF2 and EGF (ax0047 and ax0047X, Axol Bioscience, UK), and kept in a standard humidified air incubator (5% CO_2_, 20%O_2_, 37°C) (full cell culture protocol, as well as further information on each cell line available in the Supplementary material). Each MFD was coated with the same combination of PLO and L-15 laminin and seeded with 1.1 × 10^5^ NSCs (37,000 cells per cell chamber), from which point synchronous differentiation and maturation of the NSCs into cortical neurons was carried out until day 15, using an NSC reagent bundle and media (ax0101, Axol Bioscience, UK) in accordance with the manufacturer’s protocol.

### Transient excitatory stimulation of multi-nodal neural networks using kainic acid

Fifteen days post seeding, the multi-nodal neural networks were stimulated with kainic acid (KA, ab144490, Abcam, UK) and subsequently investigated with live staining assays. KA is a potent neuronal excitant, stimulating ionotropic glutamate receptors. KA-induced excitotoxicity has been used to model neurodegeneration, and depending on concentration, KA-stimulation can lead to increased production of reactive oxygen species (ROS), mitochondrial dysfunction and apoptosis (Wang et al., 2005). KA (10 μM) was applied to the top cell chamber for 30 min, after which all cell chambers were washed three times with Dulbecco’s phosphate buffered saline (PBS, 806544, Sigma Aldrich, US) and resupplied with media. A flow barrier created by a 10 μL media level difference between the stimulated chamber and the non-stimulated chambers was used to confine the KA to the top cell chamber only. The same procedure was carried out for each cell line using PBS as a sham stimulation. Following the 30-min KA stimulation targeting the neurons in the top cell chamber of the multi-nodal neural network, live microscopy of fluorescently labeled ROS production confirmed that the stimulation was successfully confined to the target chamber (Supplementary Figures S1A–E). Furthermore, 24 h later both stimulated and sham-stimulated (PBS) neural networks from each group were assessed using a live/dead viability assay kit, demonstrating that the stimulation was sublethal (Supplementary Figures S1F–H).

### ROS and live/dead assays

A total ROS assay kit (ex/em 644/665) (C10422, Thermo Fisher Scientific, US) fluorescently labeling ROS production was applied to verify a cellular response and the confinement of the stimulation by fluorescence microscopy (EVOS FL auto 2, Invitrogen, California, US) where the microscope was set to image simultaneously in each of the MFD chambers every 5 min for 1 h immediately following KA stimulation. Analysis of ROS expression was performed using the Fiji plugin Particle analyzer. 24 h post KA stimulation, a live/dead viability/cytotoxicity kit (MP03224, Invitrogen, US) was applied to the neural networks in the MFD. 0.8 μL Ethidium homodimer-1 (2 mM in DMSO/H_2_O 1:4) and 0.4 μL Calcein AM (4 mM in anhydrous DMSO) was diluted in 2 mL PBS and applied to all chambers for 15 min in 37°C. The fluorescently labeled multi-nodal neural networks were then washed with PBS and imaged (EVOS FL auto 2). The two-channel fluorescent images from the viability assay were merged, adjusted for brightness/contrast, and the cells manually counted using the Cell counter Fiji-plugin. The area close to the active zone in the MFD was selected for analysis as this area showed better separation of the signals due to a consistent lower cell density across all conditions. Two areas from each cell chamber were counted in two separate multi-nodal neural networks (>2000 cells/network) for each condition (KA- vs. sham-stimulated) in each group (control vs. LRRK2) (Supplementary Figure S1H).

### Immunocytochemistry

24 h post KA stimulation (or PBS sham stimulation), multi-nodal neural networks from both the isogenic control and LRRK2 groups were fixed and used for immunocytochemistry assays to assess the neurite and spine morphology. For fixation, 2% paraformaldehyde (PFA, 158127, Sigma Aldrich, US) was applied for 15 min followed by 4% PFA for 10 min and 3 × 15-min washes at room temperature (RT). Blocking solution consisting of PBS with 5% normal goat serum (NGS) and 0.3% Triton-X (HFH10, Thermo Fisher Scientific, US) was applied for 2 h at room temperature and was followed by overnight incubation in primary anti-body solution (PBS with 1% NGS, 0.1% Triton-X) in 4°C. The following antibodies were used: Rabbit anti-Piccolo antibody (1:400) (ab20664, Abcam, UK), mouse-anti PSD95 (1:200) (ab13552, Abcam, UK), rabbit anti-CaMKΙΙ (1:250) (ab134041, Abcam, UK), rabbit anti-GRIK5 (1:200) (PA-5-41401, VWR, US), rabbit anti-Glutamate receptor 1 (AMPA) (1:500) (ab109450, Abcam, UK), mouse anti-MAP2 (1:400) (131,500, Thermo Fisher Scientific, US), mouse anti-beta ΙΙΙ tubulin (1:400) (ab119100, Abcam, UK), rabbit anti-total alpha synuclein (ab131508, Abcam, UK), mouse anti-mitochondria (ab3298, Abcam, UK), and chicken anti-neurofilament heavy (1:1000) (ab4680, Abcam, UK). The multi-nodal neural networks were then washed 3 × 15 min in PBS, and incubated for 3 h in secondary antibody solution (PBS with 1% NGS, 0.1% Triton-X) in the dark, at RT. A combination of Alexa Fluor™ 488, 568, 647 secondary antibodies (Thermo Fisher, MA, US) were used at a dilution of 1:1000. CytoPainter Phalloidin 647 (1:500) (ab176759, Abcam, UK) was added for the final 20 min of incubation, and Hoechst (1:10000) was added for the final 5 min before another 3 × 15-min wash in PBS was conducted. Images used for quantification were taken using a Zeiss Axiovert 1A fluorescent microscope (Carl Zeiss, Germany) with a 100×/1.3 oil objective or a Zeiss (510 META Live) confocal laser scanning microscope with a 63×/1.4 oil objective. ImageJ, MATLAB and PowerPoint were used to post-process the images.

### Image analysis

The fluorescence images of Piccolo-immunolabeled neurites were analyzed using a semi-automated process implemented in MATLAB and Fiji to count the number of neuritic boutons. Most boutons were counted in an automated fashion in MATLAB. Top-hat filtering and contrast enhancing using adaptive histogram equalization was applied. The contrast-enhanced images were binarized using Otsu thresholding; a threshold selection method where images are separated into two intensity classes from gray-level histograms, namely foreground and background (binarization), and salt noise in the binarized images was removed by median filtering. Neurite fragments were then joined by morphological closing, and any remaining small fragments were removed by hole filling. Thinning was then applied to obtain a skeleton of the neurites in the image. From this thinned image, endpoint detection was used to obtain a preliminary bouton count. The endpoint-labeled images were visually inspected and any missed boutons were manually counted and added to the final results. The area covered by the neurites was calculated by means of the particle analyzer after binarization with Otsu thresholding. Together, the boutons counted divided by the area covered with neurites created a ratio used for statistical analysis. For the measure of co-occurring Piccolo and PSD95 immunolabeling, an automatic threshold was applied for each channel (Otsu for Piccolo and Triangle for PSD95) in Fiji, the thresholded areas selected as ROIs, and areas containing both ROIs were selected for particle analysis. A cut-off at 15 μm was set as an upper limit, and the number and average size measurement from each image were used for statistical analysis.

### Dynamics of neuritic mitochondria

To investigate the distribution and dynamics of mitochondria in LRRK2 G2019S and isogentic control neural networks, 0.1% Tetramethylrhodiamine (TMRM, T668, Invitrogen, US) was applied for 30 min at 37°C to all MFD chambers, rinsed in PBS, and imaged using a Zeiss 510 META Live confocal scanning laser microscope in a heated chamber (37°C). This dynamic mitochondria stain is readily sequestered by active mitochondria with intact membrane potentials. As a baseline measure, three image series were taken every 10 min, where an image was taken every second for 1 min.

The number and size of the TMRM-labeled mitochondria within the axonal tunnels were extracted using a simple image analysis pipeline implemented in MATLAB R2018. First, nonuniform background illumination was removed by applying a top hat filter, before image segmentation by Otsu thresholding was performed. The 8-connected components were then extracted from the resulting binary image. Artifacts at the edges of the images, which tended to be large and elongated, were removed by eliminating components if their size exceeded 5 μm^2^ or eccentricity exceeded 0.995. The number of mitochondria was then extracted as the number of remaining 8-connected components in the image. The size of each detected mitochondrion was computed from the number of pixels comprising each as-detected component. Additional analysis of fixed samples from this experiment were analyzed in Fiji using ROI manager. Two channel 100× images of fluorescent mitochondria and total alpha synuclein in the neural networks were binarized by Otsu thresholding, and the area covered by the fluorescence in each channel was selected and measured using the ROI manager. A ratio of the area covered by mitochondria/total alpha synuclein was calculated and used for statistical analysis in Prism8 (GraphPad, California, United States).

To investigate the mitochondrial motility, a semi-automated image analysis process implemented in MATLAB R2020a. As stated above, each image series used for the analysis consisted of one image taken every second for 1 min, and these image series each spanned four microtunnels. Four kymographs were created from each image series by isolating each microtunnel in the series. Each row of pixels in the kymographs represents the average luminance across the width of the tunnel, and these rows were stacked to show the time series progression of the luminance along the vertical axis. Filtering was applied to reduce the presence of vertical stripes in the kymographs, which represent stationary mitochondria. Motile mitochondria were then identified as slanted lines in the kymographs, and their speeds were calculated as the change in distance along the tunnel (horizontal axis in the kymograph) divided by the change in time (vertical axis) (Supplementary Movies S1, S2).

### Electrophysiological investigation

Using an identical MFD design interfaced with a custom-made multielectrode array (MEA) (Supplementary Figure S2), the electrophysiological activity of the multi-nodal neural networks and their response to KA or sham stimulation were recorded through the MEA2100 *in vitro* headstage, interface system and software suite (Multi Channel Systems; Reutlingen, Germany). The networks were recorded for 7 min durations immediately before KA stimulation (baseline), during stimulation, and at 24 h post-stimulation (10 kHz sampling rate, Butterworth filtering, 2nd order, 300–3,000 Hz band pass, with a spike detection threshold of-5 standard deviations from the total signal median). For each of the two experimental groups (isogenic control neural networks and LRRK2 neural networks), 6 MEA-interfaced multi-nodal neural networks were recorded and analyzed (3 PBS sham stimulated, 3 KA stimulated). All raw data recordings are published in Mendeley Data Repository.^1^

### Post-processing and analysis of electrophysiological data

Electrophysiological data analysis was performed with NeuroExplorer 4 (Nex Technologies, Colorado, United States) and MATLAB (MathWorks 2020, Massachusetts, United States). Following filtering and spike detection, the spikes were binned (1 ms) and the electrodes ordered according to chamber or channel of origin. Mean firing rates (MFRs) were estimated across conditions and recording time points as spikes per second. As a measure of functional connectivity, the cross-correlation was estimated through Pearson’s correlation coefficient r for concurrent spiking. First, inactive electrodes (< 10 spikes per recording) were excluded before a pairwise comparison was performed between all electrodes at a maximum signal lag of 100 ms. Non-significant correlations (*p* > 0.001) were excluded to reduce the number of spurious connections. The correlations between electrodes were finally selected as peak lag (i.e., the correlation between two electrodes could for example peak at 52 ms delay, where this correlation would then be the final connection weight). The total network correlation was computed as the mean *r* across electrodes per recording.

### Statistical analyses

For both the LRRK2 group and isogenic control group the number of independent multi-nodal neural networks is 6, for both regular MFDs and MEA-coupled parallels. All statistical analyses was performed and graphed using Prism8 (GraphPad, California, United States), and the exact statistical test used in each case is stated in the results section. Normally distributed datasets were analyzed using parametric tests, while non-parametric tests were used if the assumption of normality was violated. n = neural networks, n = images (neural networks). For the mitochondrial analysis n = axon tunnels (neural networks), n = mitochondria (neural networks). In this study, the isogenic control neural networks represent what is considered as healthy development and function, and the deviation from it a sign of pathology.

## Results

### Establishment of multi-nodal neural networks in microfluidic devices

**Figure 1:**
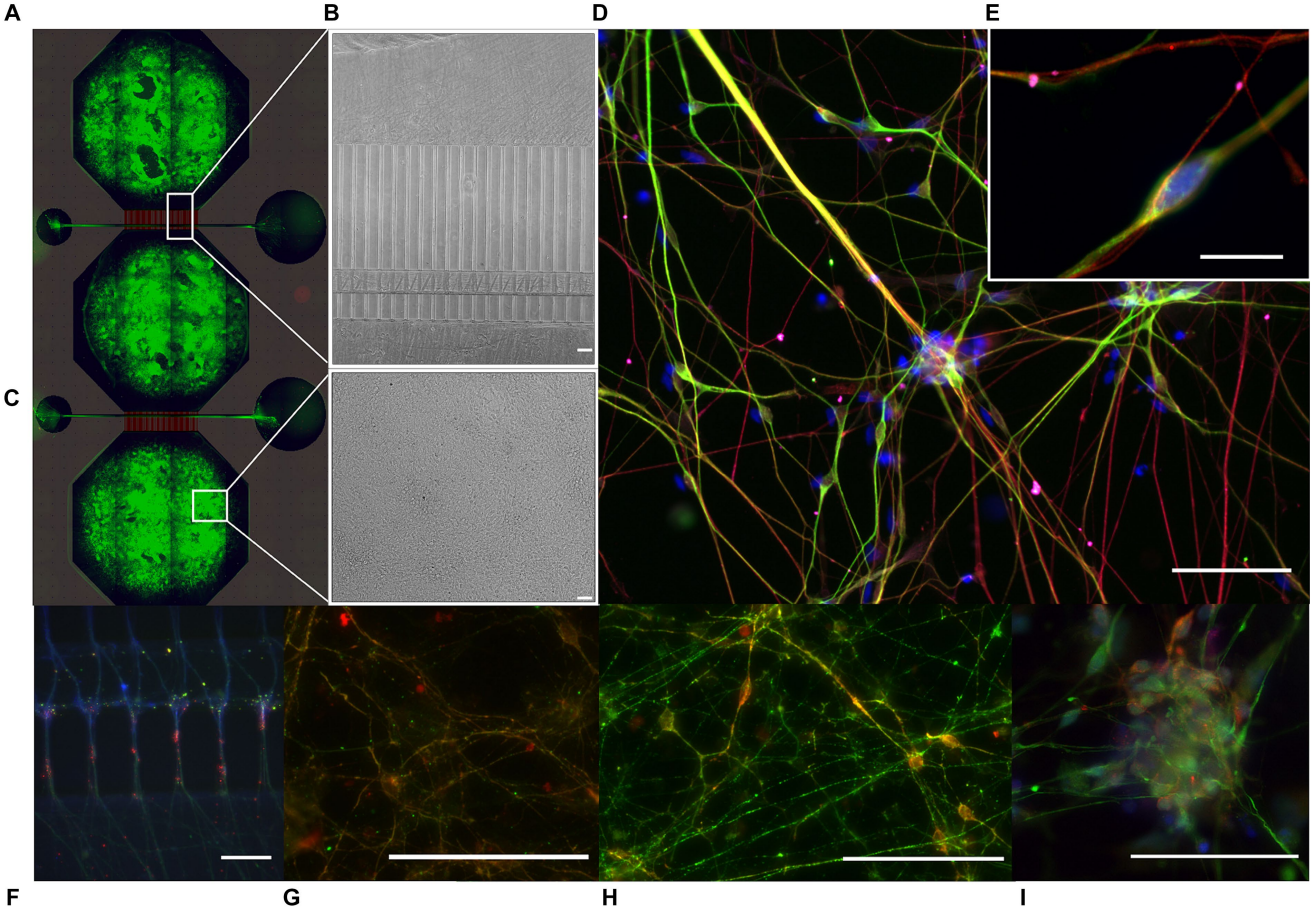
Immunocytochemistry of multi-nodal neural networks in microfluidic devices. Following 15 days of NSC differentiation and maturation, immunocytochemistry indicated the presence of mature neural networks in the microfluidic device (MFD). **(A)** Tiled image of a fluorescently labeled cortical multi-nodal neural network within a MFD, overlaid by a schematic of the design. **(B,C)** Brightfield images of the developing multi-nodal neural network, showing the area containing the axon tunnels, synaptic compartment, and cell chamber, respectively. **(D)** Fluorescently labeled LRRK2 G2019s network with markers for neurons (MAP2, green), neuron specific microtubules (beta-ΙΙΙ tubulin, red), and kainic acid receptors (GRIK5, magenta) together with the counterstain Hoechst (blue), with **(E)** showing equivalent markers in a healthy isogenic control neural network (10 μm scale bar). The remaining images show the neural networks fluorescently labeled with markers for **(F)** neurons (MAP2, red) expressing calmodulin-dependent protein kinase 2 (CaMKΙΙ, green), **(G)** with presynaptic vesicles (Piccolo, green), postsynaptic densities (PSD95, red) and F-actin (Phalloidin, blue) expressed in the axon tunnels and synaptic area, **(H)** neuronal specific microtubules (beta-ΙΙΙ tubulin, red) together with CaMKΙΙ (green), and **(I)** neurofilament heavy (green) together with AMPA receptors (red) and Hoechst counterstain. 50 μm scale bars.

Neural networks derived from both the isogenic control and LRRK2-mutated NSCs were successfully established using the MFDs (Figure 1, see also Supplementary Figures S2, S3 for detailed microfluidic device layout). Following 15 days of differentiation and maturation, the neural networks form both groups showed positive immunolabeling of neurons (MAP2), with neuron-specific microtubules (beta-ΙΙΙ tubulin) and mature axons (neurofilament heavy) containing both pre- and post-synaptic elements (Piccolo and PSD95, respectively), as well as expressing calmodulin-dependent protein kinase II (CamKII), a marker related to synaptic connectivity and long-term potentiation. Importantly, both kainic acid receptors (GRIK5) and AMPA receptors (GluR1) were also present (Figures 1D,E,I).

### LRRK2 G2019S neurons self-organize into networks with aberrant neuritic outgrowth and mitochondrial dynamics

**Figure 2:**
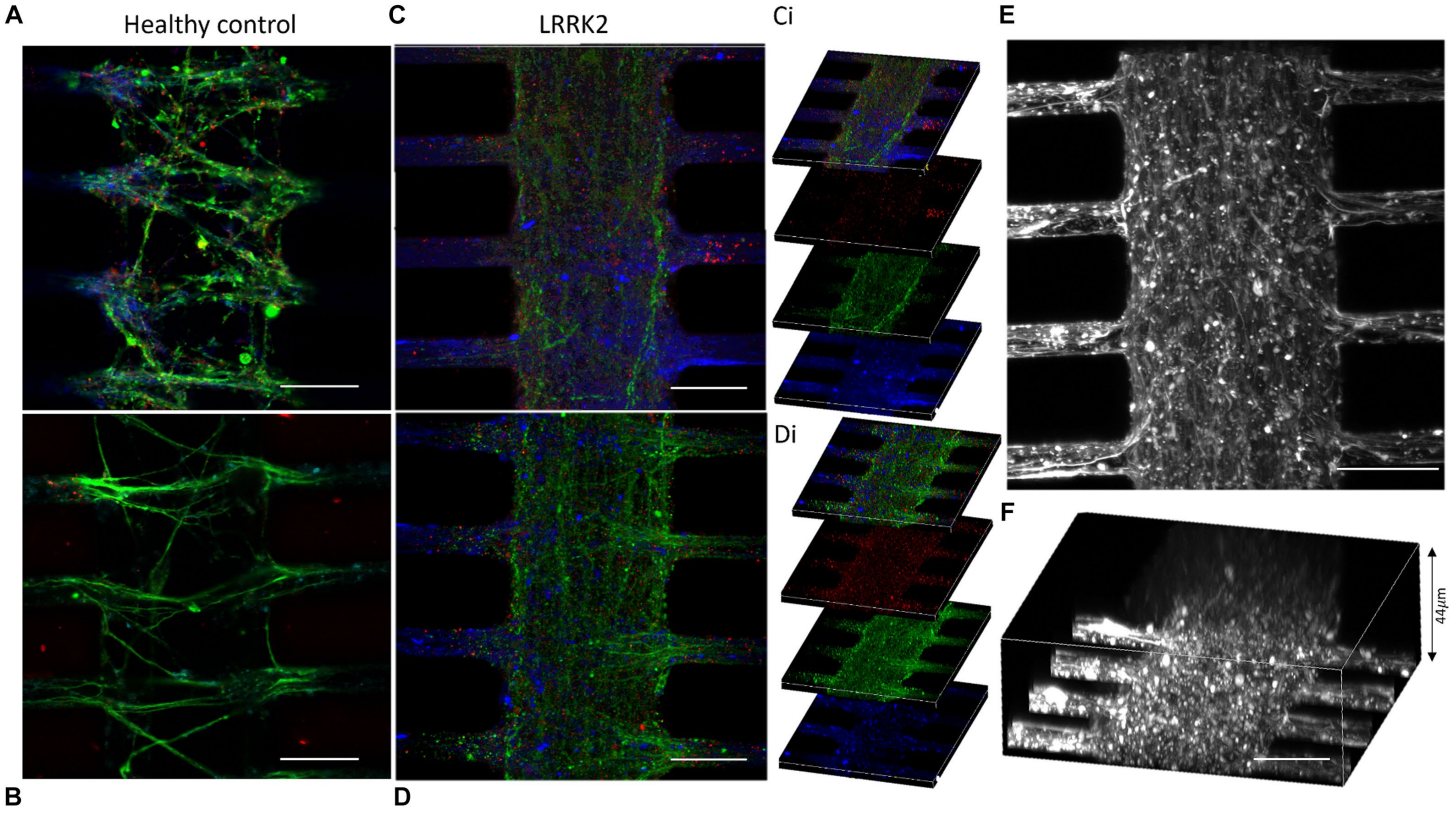
Outgrowing neurites overcrowding the synaptic compartments of the LRRK2 G2019S multi-nodal neural networks. **(A,B)** Show control neural networks fluorescently labeled with pre- and postsynaptic markers (piccolo, green and PSD95, red, respectively) and a cytoskeletal f-actin filament marker (Phalloidin, blue). **(C,D)** Display equivalent images from LRRK2 neural networks with neurites overcrowding the synaptic compartment. **(Ci,Di)** Show 10 μm thick z-stacks compiled into **(C,D)**, respectively. **(E,F)** Show LRRK2 neural networks labeled with phalloidin, where the volumetric sideview **(F)** demonstrates a neurite bundle thickness of 44 μm. 30 μm scale bar.

The morphology of the neurites in the multi-nodal neural networks was evaluated by fluorescent labeling of cytoskeletal f-actin filaments (Phalloidin), and pre- and post-synaptic markers (anti-Piccolo and anti-PSD95 antibodies). Here, neurites were observed overcrowding the synaptic compartments of the LRRK2 neural networks. These thick bundles of neurites required image stacks up to 44 μm to capture the entirety of the structures. By comparison, the neurites in isogenic control networks required 15 μm stacks (Figure 2). Within the synaptic compartments of the LRRK2 G2019S neural networks neurites were observed crossing perpendicular to the axonal tunnels (Figure 2; Supplementary Figure S3). Some neurite bundles were observed to protrude into the synaptic inlet and outlet chambers, suggesting random neuritic outgrowth (Supplementary Figure S4). The neurites of the isogenic control neural networks were less densely packed, did not protrude into the inlet and outlet chambers, and displayed prominent directional, fasciculated outgrowth (Figure 2). These differences in self-organized network morphology and neurite outgrowth profile, was consistently observed across all assays throughout the experiment, highlighting differences between the groups in terms of inter-nodal structural connectivity (Supplementary Figure S3).

**Figure 3:**
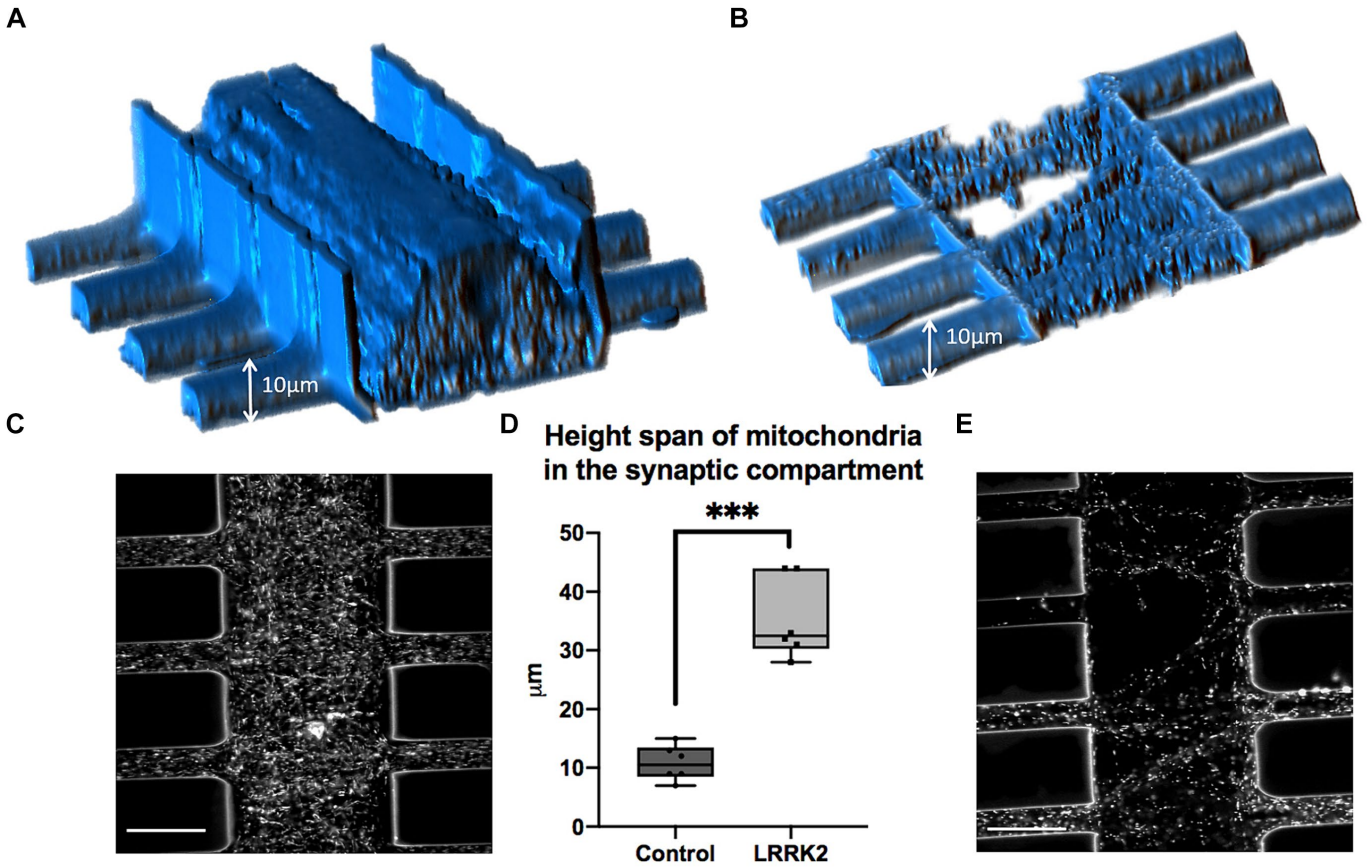
Volumetric comparison of LRRK2 and control neurites crossing the synaptic compartment. **(A,B)** Show volumetric figures obtained from z-stack images of neurites after TMRM labeling. **(A)** LRRK2 neurites (height = 44 μm). **(B)** Healthy control neurites (height = 9 μm). **(C,E)** Show single slices within a z-stack from **(A,B)**, respectively. **(D)** Graph showing a three-fold mean height measure of volumetric figures (*p* < 0.0001). 30 μm scale bar.

Mitochondria were labeled with TMRM and visualized live in the multi-nodal neural networks. Image stacks (single z-stacks) containing all of the TMRM-labeled mitochondria within a representative segment of the synaptic compartment were obtained for both the LRRK2 (*n* = 6) and isogenic control (*n* = 6) neural networks. In Figures 3A,B volumetric figures produced from the TMRM z-stacks reflects and corroborates the qualitative observation made in Figure 2 and Supplementary Figure S3 of highly distinct network morphologies, with significant differences in neurite profiles between the groups. Height measures calculated from the z-stacks showed a statistically significant difference between the LRRK2 and control neural networks (two-tailed, independent samples *t*-test, t_10_ = 7.96, *p* < 0.0001), with the LRRK2 neural networks containing synaptic compartment mitochondria spanning on average over 3 times the height of the control neural networks (mean_LRRK2_ = 35.38 μm vs. mean_control_ = 10.83 μm) (Figure 3D).

**Figure 4:**
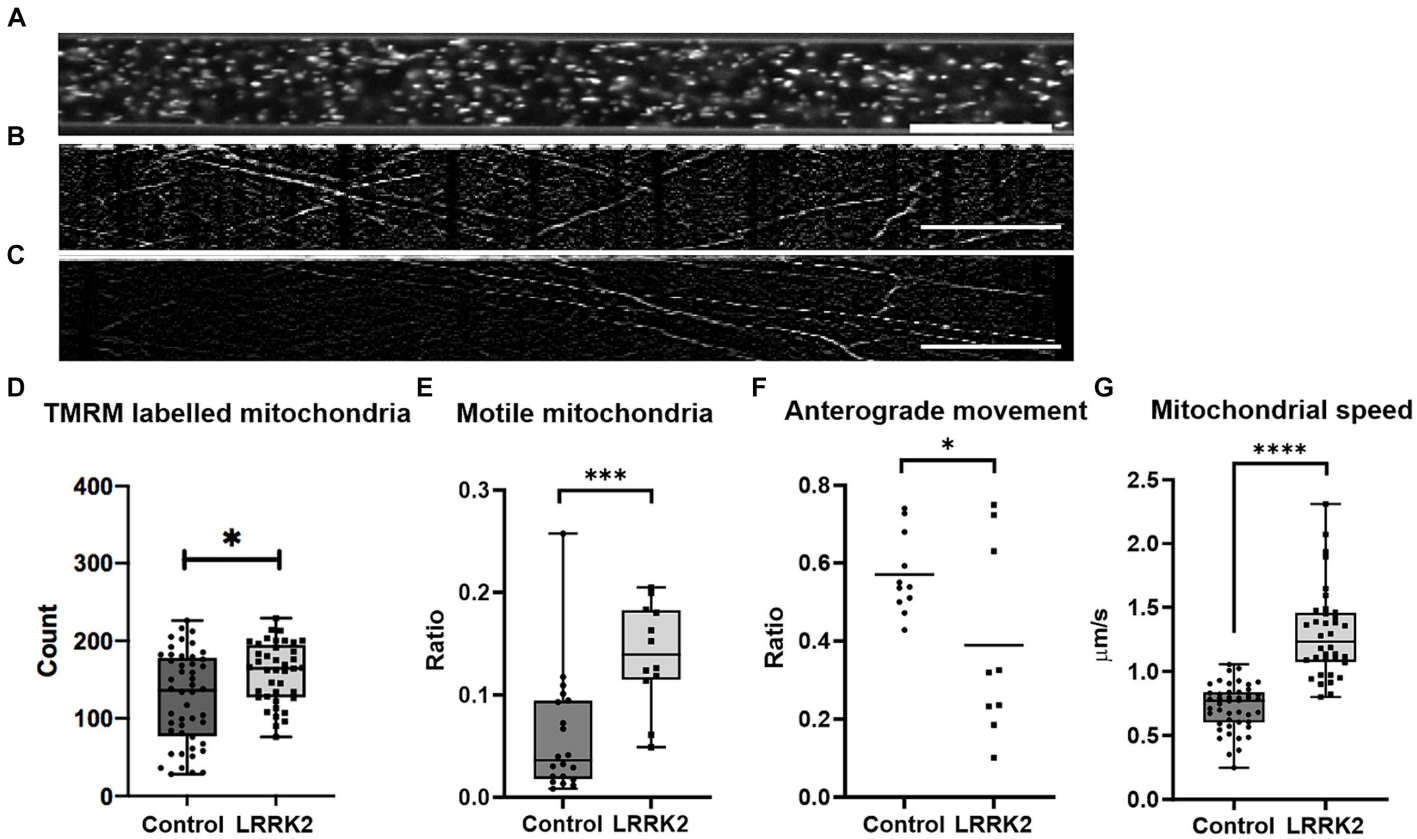
Comparison of mitochondrial distribution and dynamics in LRRK2 vs. healthy control neurites. **(A)** Shows an axonal tunnel containing TMRM labeled mitochondria. **(B,C)** representative kymographs from a healthy control and LRRK2 neural network, respectively. Slanted lines indicate motile mitochondria. **(D)** Difference in number of TMRM labelled mitochondria (*p* = 0.0114), in **(E)** ratio of motile mitochondria within the axon tunnels (*p* = 0.0005), and in **(F)** directionality (*p* = 0.0388) between TMRM-labeled mitochondria in LRRK2 vs. healthy control neurites. **(G)** Difference in speed of individual motile mitochondria of LRRK2 and control neural networks (*p* < 0.0001). *p < 0.05, ***p < 0.0005, ****p < 0.00005, (box and whiskers are minimum to maximum, all data points included), and n = axon tunnels (neural networks) for all panels, except for **(G)** where n = motile mitochondria (neural networks). Speed = Δhorisontal axis/Δ vertical axis, 20 μm scale bar.

For each neural network, the fluorescently labeled mitochondria contained within 145-μm long segments (Figure 4A) of different axonal tunnels were used for further analysis. Here, the groups were found to contain a small, but statistically significant difference in number of mitochondria (Mann Whitney *U* = 659.6, *n*_LRRK2_ = 40(5), *n*_control_ = 48(6), *p* = 0.0114,), with a median count of 164.5 mitochondria per tunnel for the LRRK2 G2019S compared to 136 for the controls (Figure 4D). However, as suggested by a supplementary investigation of fluorescently labeled mitochondria in fixed samples (Supplementary Figure S5), this difference is likely more indicative of a greater number of neurites containing mitochondria in the axonal tunnels of the LRRK2 G2019S networks rather than a greater number of mitochondria being contained within each neurite.

Furthermore, the quality of the live image series from 3 LRRK2 G2019S and 5 isogenic healthy control neural networks allowed for conversion into kymographs and was used for assessing the mitochondria dynamics within the axonal tunnels. Each kymograph was generated from 60 images (1 image/s taken consecutively for a minute) (Figures 4B,C; Supplementary Movies S1, S2 for example kymographs). Investigation of mitochondrial dynamics revealed a significantly greater ratio of motile mitochondria in the axonal tunnels of LRRK2 G2019S neural networks compared to the healthy controls (mean ratio of 0.139 vs. 0.06, *t*_30_ = 3.899, *n*_control_ = 20 (5), *n*_LRRK2_ = 12 (3), *p* = 0.0005) (Figure 4E). Furthermore, the individual motile mitochondria of the LRRK2 networks were moving at significantly greater speeds compared to those of the healthy controls (mean 1.296 μm/s vs. 0.639 μm/s, Mann Whitney *U* = 56, *n*_control_ = 46 (5), *n*_LRRK2_ = 36 (3), *p* < 0.0001) (Figure 4G). When it comes to direction of movement, the mitochondria of the control neural networks tended to be more balanced, with most tunnels displaying a 0.4–0.6 ratio of anterograde mitochondrial movement. By contrast, the mitochondria of the LRRK2 neural networks had clear directional tendencies, with movements skewed either anterogradely or retrogradely (*t*_18_ = 2.229, *n*_control_ = 11 (5), *n*_LRRK2_ = 9 (3), *p* = 0.0388) (Figure 4F). Finally, a greater percentage of tunnels in the control neural networks displayed mitochondria with short, bidirectional movements (fluctuating back and forth around the same point) (30.5%) compared to the LRRK2 neural networks (19.5%). However, this final note is stated merely as an observation, as this particular pattern of movement was not faithfully captured by the kymographs, and thus might be underrepresented.

### Transient perturbation reveals significant differences in immediate mitochondrial response, as well as in neuritic- and synaptic remodeling 24-h post perturbation between LRRK2 and healthy control neural networks

**Figure 5:**
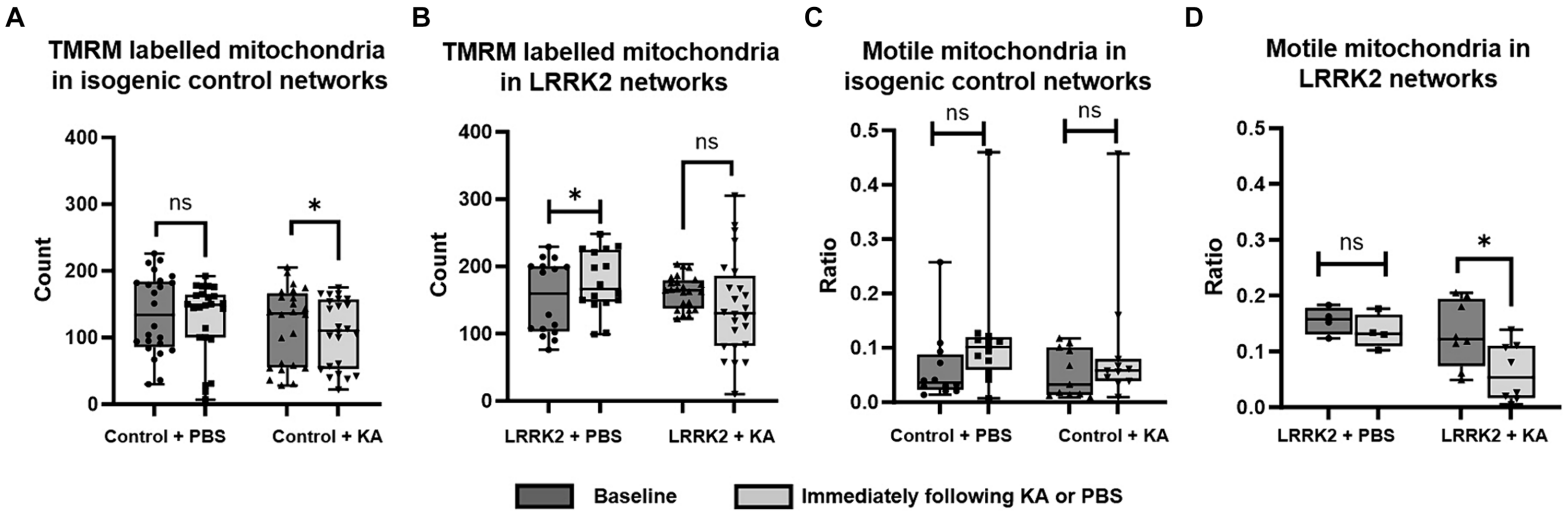
Immediate mitochondrial response following transient perturbation. **(A)** The healthy control neural networks were found to contain significantly fewer mitochondria immediately after KA stimulation compared to the baseline (*p* = 0.0432), while the LRRK2 neural networks, **(B)** showed no such difference after KA stimulation. After sham stimulation, however, the LRRK2 neural networks showed a statistically significant difference (*p* = 0.029), with more mitochondria being measured after PBS addition. **(C,D)** Show the ratio of motile vs. stationary mitochondria measured at baseline and immediately after KA or PBS addition, for the control and LRRK2 networks, respectively. No significant difference was found after PBS or KA stimulation for the control networks, while the LRRK2 networks showed a significant reduction in motile mitochondria after the KA stimulation only (*p* = 0.0155) Graphs shown in **(A–D)** are box and whisker plots (min to max bars, all data points included). * p < 0.05, *n* = axon tunnels (neural networks).

To investigate whether the number of mitochondria was influenced by a transient perturbation (KA), the TMRM labeled mitochondria contained within the axonal tunnels of both control and LRRK2 neural networks were imaged live at baseline and immediately after KA or PBS (sham stimulation) addition (Figures 5A,B). The same area of the same axonal tunnels was imaged at each timepoint for each network. A significant difference was found by Wilcoxon matched-pairs signed ranks test [pairs = 24 (3), *p* = 0.0432] for the control neural networks, with a reduced number of mitochondria measured after the KA stimulation, but not for the LRRK2 networks [pairs = 24 (3), *p* = 0.1578]. For the networks receiving sham stimulation, significantly more mitochondria were found at the section timepoint for the LRRK2 neural networks [pairs = 16 (2), *p* = 0.029], while no difference was found for the healthy control neural networks [pairs = 24 (3), *p* = 0.214]. The mitochondrial motility (ratio of motile vs. stationary mitochondria) was also assessed at these timepoints (Figures 5C,D). No significant difference was found after perturbation or sham stimulation for the control neural networks [paired *t*-test, two tailed: *p* = 0.0613, *t* = 2.084, df = 11 (3) for the PBS condition, and *p* = 0.1171, *t* = 1.715, df = 10 (3) for the KA condition]. However, the LRRK2 networks showed a significant reduction in motile mitochondria after the KA stimulation only [paired *t*-test, two-tailed, *p* = 0.0155, *t* = 3.179, df = 7 (2)].

**Figure 6:**
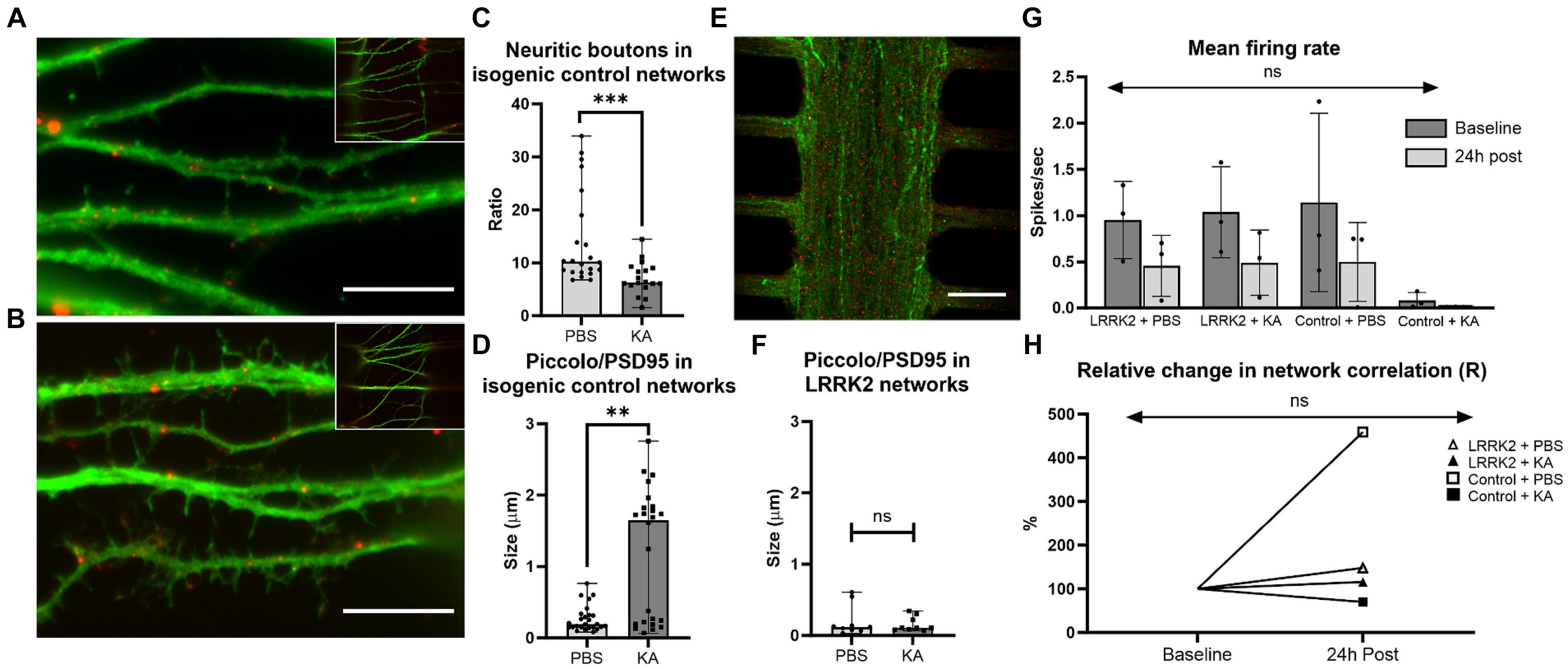
Neural network response 24 h after perturbation. **(A–F)** show data from cortical neural networks fluorescently labeled with pre- (Piccolo, green) and postsynaptic (PSD95, red) markers 24 h post KA or sham (PBS) stimulation. Control neural networks from the KA and PBS condition are shown in **(A,B)**, respectively. Similarly, **(E)** shows an LRRK2 neural network from the KA stimulated condition, illustrating the lower level of morphological detail available due to the density of neurites. **(C)** Shows a significant reduction in neuritic boutons (*p* = 0.0004) an **(D)** significant enlargement in synaptic contact size (Piccolo/PSD95 co-occurrence) (*p* = 0.0017), 24 h post KA stimulation compared to sham stimulation (PBS) in the healthy control networks. **(F)** No significant difference was found in synaptic contact size between the KA and sham stimulation condition in the LRRK2 networks. **(G)** Displays a bar graph of mean firing rate (with SD bars) measured in the MEA-coupled MFDs for each group at baseline and 24 h post perturbation. No statistically significant difference was found (*p* = 0.977). **(H)** Line graph displaying the relative change in total network correlation (Pearson’s r) from baseline to 24 h post stimulation, with no statistically significant difference found (*p* = 0.929). For **(C,D,F)** n = images (neural networks), ns *p >* 0.05, **p < 0.005, ***p < 0.0005 (median with range bars, all data points included). 10 μm scale bars.

24 h post perturbation or sham stimulation, high-magnification microscopy images of the multi-nodal neural networks fluorescently labeled with pre- and postsynaptic markers (anti-Piccolo and anti-PSD95) were used for morphological investigations of the neurites in the synaptic compartment, and for quantification of synaptic contacts (Figures 6A–F). Prominent morphological differences were observed in the healthy isogenic control neural networks following perturbation compared to the sham condition. The healthy controls had substantially fewer synaptic boutons (median_KA_ = 6.32 vs. median_PBS_ = 10.24, *p* = 0.0004), and substantially larger synaptic contact areas (median_KA_ = 1.651 μm vs. median_PBS_ = 0.1915 μm, *p* = 0.0017) after KA (Figures 6C,D). Due to the sheer volume of neurites in the LRRK2 neural networks it was not feasible to investigate the morphology of single neurites (counting the synaptic buttons) for comparison (Figure 6E). To evaluate the synaptic contact size, the bottommost image slice from each image-stack was used for quantification. No significant difference was found in the size of synaptic contact area between the KA and PBS condition (median_KA_ = 0.1060 μm vs. median_PBS_ = 0.1190 μm, *p* = 0.9506) of the LRRK2 G2019S neural networks (Figure 6F).

The electrophysiological activity of 6 multi-nodal neural networks from each group (n_control_, n_LRRK2_ = 6) was also investigated using MFDs interfaced with MEAs in parallel assays. At 15 days post seeding, electrophysiological recordings were obtained for all networks were immediately before (baseline) and at 24 h after administration of PBS or KA. As a basic functional evaluation, the average mean firing rate and total cross-correlations were calculated. The healthy isogenic Control + KA group displayed very low mean firing rates (MFRs) measures at both timepoints (MFR_baseline_ = 0.082 ± 0.249 spikes/s and MFR_24h post_ = 0.011 ± 0.012 spikes/s). However, all other groups displayed comparable baseline MFRs, within the range of 0.953–1.143 spikes/s and with standard deviations between 1.764–1.963. At 24 h after PBS or KA, all networks displayed the same trend, with lower MFRs, compared to baseline (0.393–0.499 spikes/s ± 0.921–1.019). No statistically significant difference in MFR was found by two way repeated measures ANOVA [*F*(3,16) = 0.066, *p* = 0.977] (Figure 6G).

Furthermore, the total network correlation (Pearson’s r) was calculated for each group, where the relative change in Pearson’s r between the baseline and 24 h timepoint is displayed graphically in Figure 6H. Repeated measures two way ANOVA revealed no statistically significant differences in total network correlation following either KA or sham stimulations [*F*(3, 16) = 0.149, *p* = 0.929]. Nonetheless, with the notable exception of the isogenic Control + KA group, which had a decrease in total network correlation (to 70%, from r_baseline_ = 0.013 to r_24h post_ = 0.009), all groups displayed an increase in total network correlation at the 24 h post timepoint compared to their baselines: Control_PBS_ to 459% (from r_baseline_ = 0.004 to r_24h post_ = 0.019), LRRK2_PBS_ to 148% (from r_baseline_ = 0.008 to r_24h post_ = 0.013), and LRRK2_KA_ to 114%, (from r_baseline_ = 0.006 to r_24h post_ = 0.008).

## Discussion

Studies using both *in vitro* and *in vivo* models of PD suggest that axonal dysfunction and synaptic alterations represent the earliest detectable signs of the disease (MacLeod et al., 2006; Cheng et al., 2010; Dagda et al., 2014) and that initiation of pathology at the axon terminals might signify the start of the retrograde degeneration of the neurons (Tagliaferro and Burke, 2016; Sheehan and Yue, 2018). Although extensively reported on Smith et al. (2005), West et al. (2005), MacLeod et al. (2006), Smith et al. (2006), Plowey et al. (2008), Gillardon (2009), Dachsel et al. (2010), Meixner et al. (2011), Cherra et al. (2013), Habig et al. (2013), and Sepulveda et al. (2013), the specific involvement of the LRRK2 gene in neurite process morphology remains obscure. We found the LRRK2 G2019S neural networks to have a much greater volume of neurites within the axonal tunnels and synaptic compartments, as well as random outgrowth profiles compared to the healthy isogenic control. This result was corroborated through several different imaging approaches, i.e., immunocytochemistry, bright field microscopy, Calcein-AM labeled cells, as well as live imaging of TMRM labeled mitochondria. In contrast to our findings, the specific G2019S PD-associated LRRK2 mutation has generally been found to result in a progressive reduction of neurite length and branching (West et al., 2005; Greggio et al., 2006; MacLeod et al., 2006; Smith et al., 2006; Plowey et al., 2008; Chan et al., 2011; Nguyen et al., 2011; Winner et al., 2011; Sanchez-Danes et al., 2012; Cherra et al., 2013; Reinhardt et al., 2013; Dagda et al., 2014; Qing et al., 2017), due to an increase in kinase activity (with one exception demonstrating non-impaired neurite morphology (Dachsel et al., 2010)). Moreover, knockdown and knock-out models resulting in LRRK2 deficiencies present with a progressive increase in neurite length in some studies (MacLeod et al., 2006; Dachsel et al., 2010; Winner et al., 2011; Habig et al., 2013; Sepulveda et al., 2013), while others find the opposite (Gillardon, 2009; Meixner et al., 2011).

The morphology of neurites and plasticity of synapses is affected by mitochondrial function as the availability of mitochondria is both essential and limiting for the support and maintenance of these structures (Li et al., 2004). A loss or impairment in dendritic mitochondria is a central feature of PD pathogenesis (Bose and Beal, 2016; Verma et al., 2017; Singh et al., 2019), and other studies on the particular LRRK2 G2019S variant have indicated compromised mitochondrial function (Cooper et al., 2012; Sanders et al., 2014; Hsieh et al., 2016; Schwab et al., 2017; Bose and Beal, 2019; Ludtmann et al., 2019). Here, the mitochondrial dynamics analysis showed that the LRRK2 G2019S neural networks contained about twice the ratio of motile mitochondria (mean 0.139 vs. 0.06), moving at about twice the speed (mean 1.296 μm/s vs. 0.639 μm/s), and with clear directional tendencies, compared to the mitochondria of the healthy isogenic control neural networks. Our baseline measurements thus point toward an overall higher metabolic cost of LRRK2 G2019S neural networks compared to healthy isogenic controls. These measurements are in line with the results from two other comparable studies, which also found an increase in mitochondrial motility in iPSC-derived neurons carrying the G2019S LRRK2 mutation compared to controls (Cooper et al., 2012; Schwab et al., 2017). Although their study was performed after 4–5 weeks of differentiation, Cooper et al. also reported an increase in mitochondrial velocity, with the LRRK2 mitochondria moving at about 0.7 μm/s versus 0.4 μm/s for the controls (Cooper et al., 2012). Moreover, in a study by Othonen and colleagues, RNA sequencing of microglia-like-cells (iMLCs) derived from iPSCs carrying the LRRK2-G2019S mutation showed alterations in pathways related to oxidative stress, which resulted in deficits in mitochondrial respiration (Ohtonen et al., 2023). In further support of our findings, these authors also report a higher oxygen consumption rate in LRRK2-G2019S carrying iMLCs compared to isogenic controls, resulting in higher respiration at the basal state.

Interestingly, previous studies investigating neuronal axonal transport kinetics, both *in vitro* and *in vivo* (Lewis et al., 2016), as well as in networks structured using microfluidic devices (Moutaux et al., 2018), have found mitochondrial dynamics to decrease with neural network maturation and development, and mitochondrial immobilization and stability to be important hallmarks of mature axons. Thus, the *in vitro* neural network traits displayed by the LRRK2 G2019S networks in our study are more prominent at very early time points in general network development. This might further suggest an impairment in network development, a notion supported by our observation of differential growth cone profile at very early stages of LRRK2 G2019S neuron differentiation compared to controls. Altered neural growth cone morphology and number has been found by others after knocking down LRRK2 (Habig et al., 2013). Importantly, the growth cone has pronounced influence on mitochondrial transportation, as nerve growth factor (NGF) signaling from immature axons regulates mitochondrial motility (Morris and Hollenbeck, 1993; Chada and Hollenbeck, 2003; Chada and Hollenbeck, 2004; Saxton and Hollenbeck, 2012). Furthermore, impaired axonal growth and guidance would explain what is observed as aberrant (and consequently inefficient) network wiring in the LRRK2 G2019S networks, with large numbers of neurites crossing perpendicular to the axonal tunnels in the synaptic compartment. Based on the general structure–function relationships of neural networks (Valderhaug, 2020), resulting aberrant wiring might again affect neurotransmission efficacy, causing less efficient signal propagation within the network, again affecting the overall energy status of the neuron and consequently the motility and distribution of mitochondria (Saxton and Hollenbeck, 2012).

Taken together, the baseline results indicate that the LRRK2 G2019S neurons self-organize into multi-nodal neural networks with inefficient wiring (aberrant neurite morphology) as well as increased mitochondrial dynamics, relative to the healthy isogenic control neural networks. Thus, both micro-and mesoscale baseline features point toward an overall heightened metabolic cost associated with the LRRK2 G2019S mutation, suggesting a network phenotype more vulnerable to perturbation.

The multiple-hit hypothesis of PD suggests that the development of the disease results from a combination of factors, rather than a single cause. This hypothesis aligns with the understanding that PD is a complex neurodegenerative disorder with a multifactorial etiology, encompassing genetic predispositions, environmental exposure, and their interactions may contribute to the onset and development of the disease (Carvey et al., 2006; Sulzer, 2007; Patrick et al., 2019). Based on our baseline results and the variability in disease progression in patients with LRRK2 G2019S associated PD, it is reasonable to assume that some phenotypic expression of the mutation may become apparent only following a significant or stressful challenge (Benson et al., 2018). Following the transient perturbation (KA) in our study, the healthy isogenic control neural networks revealed greater responses in almost all measures: a significant immediate reduction in number of neuritic mitochondria, as well as a more prominent modification in neurite morphology and synaptic remodeling, and the only measured decrease in total network correlation, 24 h post stimulation, relative to the LRRK2 neural networks. The mitochondrial motility was the only measure in which the LRRK2 neural networks displayed a greater response, with a significant reduction in mitochondrial motility immediately following perturbation, while the control neural networks showed no change.

The highly significant difference found in neurite morphology in the control neural networks with a retraction/reduction of boutons observed in the synaptic chambers 24 h post KA stimulation, suggests neurite remodeling in response to the transient perturbation. At the same time, the number of synapses (co-occurrence of pre- and post-synaptic markers) in the control neural networks was not significantly altered, but the size of their overlapping area was, with much larger synaptic areas measured 24 h after overexcitation. This rapid activity-dependent alteration in dendritic spine morphology is likely an expression of a regular mechanism for converting short-term synaptic activity to long term lasting changes in connectivity and function. In line with our baseline measurements, the size range of the postsynaptic density is usually within 0.2–0.5 μm, and can be localized to both spiny and non-spiny structures. Furthermore, this area contains both the kainate and AMPA receptors (Sheng, 2001), i.e., glutamatergic receptors targeted by our stimulation. During synaptic plasticity, the PSD increases in size in response to potentiation events, and the glutamate receptors contained within can be modulated by neural activity on a timescale from minutes to weeks (Sheng, 2001). In contrast to the observed structural changes in the control neural networks, no significant alteration in synaptic number or size was found in the synaptic compartment of the LRRK2 mutated neural networks 24 h after KA stimulation, suggesting impaired synaptic plasticity.

Importantly, this goes hand in hand with our electrophysiology results, where the KA-stimulated isogenic control neural networks were the only ones to display a relative decrease in total network correlation at the 24 h post timepoint as well, while all other conditions displayed a relative increase. Measures of MFR were comparable at all analyzed timepoints. This is in line with the findings of another highly relevant study, where the average neuronal activity *per se* was found not to be sufficiently informative to reveal disease-related alterations (Carola et al., 2021). As the transient excitatory event indiscriminately excites related connections that are both functional and non-functional, and likely produces activity-dependent synaptic modifications at both local and distal network sites (Bi and Poo, 2001), a decrease in total correlation could be expected after a “healthy” plastic network response to such an event.

Furthermore, other studies have found increased vulnerability to oxidative stress, higher levels of mtDNA damage, and impaired mitochondrial movement as a result of the G2019S mutation, indicating compromised mitochondrial function (Cooper et al., 2012; Sanders et al., 2014; Hsieh et al., 2016; Schwab et al., 2017; Bose and Beal, 2019). Our baseline measurements indicated mitochondrial activity of greater metabolic cost for the LRRK2 neural networks, which in turn would make these networks more vulnerable to perturbation. As already noted, alterations in mitochondrial function in turn affect the plasticity of synapses and morphology of neurites (Li et al., 2004). The energy status of the neuron greatly affects the motility and distribution of mitochondria (Saxton and Hollenbeck, 2012), and a relocation of the mitochondria toward either the soma or the presynaptic terminal during or following an overexcitation event is to be expected as this corresponds to the locations of greatest metabolic demand at the time. This fits with our investigations, where a reduction in number of mitochondria was found in the neurites of the control neural networks immediately following the induced excitatory event. The motility ratio of these mitochondria in turn remained unchanged, likely reflecting a regular response with mitochondria being recruited to other areas with greater metabolic demand. The LRRK2 neural networks on the other hand showed the opposite result, with no change in number of mitochondria and a significant decrease in mitochondrial motility immediately after the excitatory event. This fits well with our finding of substantial neurite and synaptic remodeling in the control neural networks only, as the mitochondria of the LRRK2 neural networks seemingly become less mobile in response to the perturbation rather than being efficiently recruited for energy metabolism and remodeling at the stimulated synapses. This also aligns well two recent *in vivo* studies, where the LRRK2 G2019S mutation was linked to impaired experience-dependent plasticity (Matikainen-Ankney et al., 2018), abnormal synaptic changes and a lack of adaptive change in intrinsic excitability (Guevara et al., 2020), in response to social stress.

The reduced motility and unaltered number of active mitochondria observed in the neurites of the LRRK2 neural networks could be a consequence of the aberrant structural network wiring, with altered signal propagation leading to impaired recruitment of mitochondria, and/or it could be due to impaired mitochondrial transport and/or function. Nevertheless, together, the baseline elevated metabolic cost and the lack of both structural and functional network alterations following perturbation, produces a network seemingly resilient to change, associated with the LRRK2 G2019S mutation. In the long term, this inefficient and energy-demanding phenotype renders the network more vulnerable.

Although graph-theory based analyses were beyond the scope of the current study, our findings are highly relevant in the context of other studies where this type of analysis was applied to either electrophysiology or fluorescent calcium imaging data. Exaggerated small-worldness, in particular, is a network structure associated with high metabolic cost, and inefficient, noisy information transfer between network regions (Bassett and Bullmore, 2017). Prominent small-world organization has been identified to underly the metabolic patterns and pathological alterations in brain network structure and function of PD-patients using neuroimaging data (Niethammer and Eidelberg, 2012; Ko et al., 2018; Schindlbeck and Eidelberg, 2018; Schindlbeck et al., 2019). Additionally, recent findings by our group using *in vitro* electrophysiological recordings from human neural networks with inherent neurodegenerative pathology show that small-world propensity associates with increased metabolic demands (Fiskum et al., 2024). This renders neural networks with predisposing genetic mutations particularly vulnerable to neurodegenerative processes, which can be precipitated by transient external perturbation. Furthermore, when compared to GBA variants of PD, the phenotypically slower progression of disease at early stages in LRRK2-PD can be explained by the gain of functional connections found in central network cores (linking the cerebellum and putamen), which could compensate and provide robustness against early symptomatic expression in prodromal stages (Schindlbeck et al., 2019). In line with this, in a relevant *in vitro* study by Carola and colleagues (Carola et al., 2021), information theory analysis was applied to fluorescence calcium imaging data. It showed that LRRK2 PD neural networks to have large, strongly linked functional communities, with lower average connectivity, ultimately producing a tendency toward greater synchrony. Thus, the overall network resilience to change in response to perturbation displayed by the LRRK2-G2019S neural networks in our study, compared to the healthy controls, reflects both underlying impairments and compensatory mechanisms.

## Summary

In summary, our study points toward an overall heightened metabolic demand, with aberrant morphology and mitochondrial dynamics, related to the G2019S LRRK2 mutation. Furthermore, these alterations seem to cause a network resilience to perturbation at early time points, as transient excitation revealed a lack of neurite remodeling- and synaptic plasticity response in LRRK2 G2019S mutated neural network compared to healthy isogenic controls. Whether such a resilience is adaptive or maladaptive, compensatory or pathological might depend on the experimental timeframe, and might cause different functional outcomes depending on PD disease stage. Thus, advanced multidisciplinary approaches such as ours, where several relevant methodologies are combined, are needed if we are to progress the field. The ability to recapitulate the relevant structural and functional dynamics of these networks at the microscale and mesoscale level opens up for entirely new avenues in modeling PD, and by the same token, other neurodegenerative diseases, including amyotrophic lateral sclerosis and Alzheimer’s disease. As such, the advanced modeling approach and new findings presented in this study are highly relevant in the quest for elucidating underlying disease mechanisms. Equally importantly, the relevant insights are fundamental for formulating new hypotheses, as well as identifying time window(s) and mode(s) of therapeutic intervention, with a view to clinical translation.

## Future perspectives

Interestingly, in a recent seminal study using alpha-synuclein seed amplification assay (SAAs) to diagnose PD with a staggering 93% accuracy, the LRRK2 PD subgroup had one of the lowest proportions of positive SAA test (67.5%) (Siderowf et al., 2023). This finding echoes the 1/3 frequency of LRRK2 PD individuals reported to lack alpha-synucleinopathy (Lewy pathology) in post-mortem studies (Kalia et al., 2015; Schneider and Alcalay, 2017), and represents a peculiarity in disease etiology which warrants further elucidation. In this context, we have shown in a previous study that this approach can be used to investigate the early functional changes associated with alpha-synuclein proteinopathy in engineered human neural networks (Valderhaug et al., 2021). It is thus highly feasible and relevant to combine such advanced multidisciplinary approaches to investigate the micro-and mesoscale aspects of LRRK2 human neural networks with a focus on the effect and development of alpha-synucleinopathy in the future. Furthermore, analogs of the validated PD network biomarker – PDRP (Parkinsons disease related pattern) (Niethammer and Eidelberg, 2012) and PDCP (Parkinsons disease cognitive pattern) (Schindlbeck and Eidelberg, 2018)– could be applied to the electrophysiological data using graph theory, and utilized to investigate disease specific network patterns, such as small-worldness (Bassett and Bullmore, 2017; Ko et al., 2018; Schindlbeck et al., 2019; Valderhaug et al., 2019; Valderhaug, 2020; Fiskum et al., 2024), in advanced cellular models based on engineered neural networks. Furthermore, fluorescence imaging can be applied to enable to visualize neuronal activity for functional characterization in such disease models. Such an approach was successfully applied in the aforementioned study by Carola and colleagues (Carola et al., 2021). Using information theory to analyze calcium activity data, revealed distinct activity patterns separating iPSC derived neuronal cultures from those of LRRK2 PD patients from isogenic controls and healthy donors. Furthermore, combination of calcium imaging with MEA-based electrophysiology can in future studies provide a more comprehensive profile of network dynamics at high spatial and temporal resolution, respectively. Such modeling approaches may thus provide a means to pinpoint structural and functional network sources that might be causally related to phenotypic variation, for instance in treatment response.

## Data availability statement

The datasets presented in this study can be found in online repositories. The names of the repository/repositories and accession number(s) can be found in the article/Supplementary material.

## Ethics statement

The human cell lines utilised in this study are commercially available and their use is regulated by a Material Transfer Agreement between the supplier and NTNU. All cell work was carried out in GMO/GMM approved Biofacility level 2 laboratories. Ethical approval was not required for the studies on humans in accordance with the local legislation and institutional requirements.

## Author contributions

VV: Conceptualization, Formal analysis, Investigation, Methodology, Visualization, Writing – original draft, Writing – review & editing. OR: Data curation, Formal analysis, Investigation, Methodology, Writing – original draft, Writing – review & editing. RW: Conceptualization, Investigation, Methodology, Writing – original draft, Writing – review & editing. KH: Formal analysis, Methodology, Writing – original draft, Writing – review & editing. SN: Supervision, Writing – original draft, Writing – review & editing. AS: Conceptualization, Project administration, Supervision, Writing – original draft, Writing – review & editing. IS: Conceptualization, Methodology, Project administration, Supervision, Writing – original draft, Writing – review & editing.

## Footnotes

^1^https://doi.org/10.17632/dnjv26msvk.4; https://doi.org/10.17632/92568tpp39.4
