# Supplementary material for: Micro-and mesoscale aspects of neurodegeneration in engineered human neural networks carrying the LRRK2 G2019S mutation
Source: Front Cell Neurosci. 2024 Apr 5;18:1366098. doi: 10.3389/fncel.2024.1366098 (PMC11026646; doi:10.3389/fncel.2024.1366098)
Supplement: Supplementary file 1 [file Data_Sheet_1.PDF]

Supplementary figures

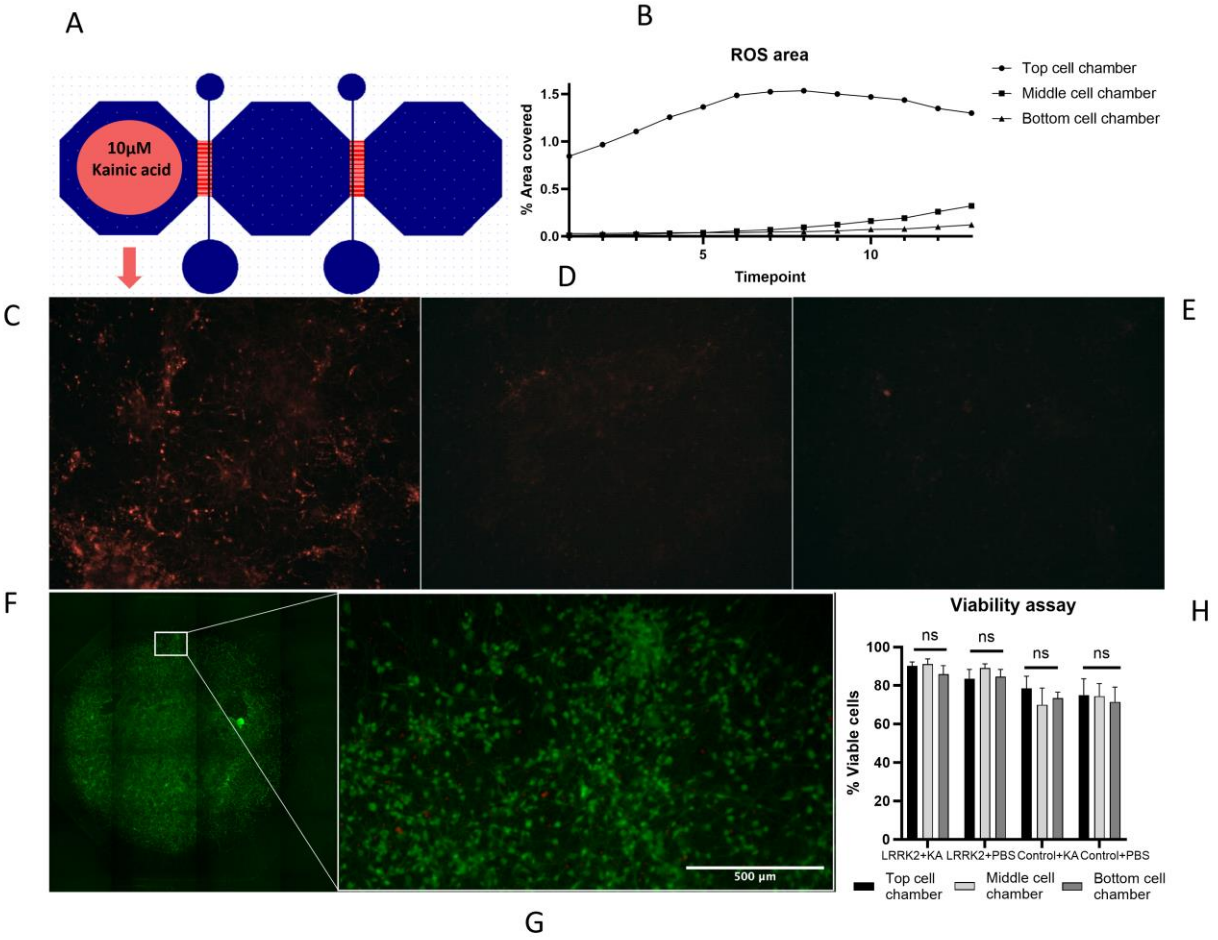

Supplementary Figure 1. Confined, transient perturbation with kainic acid (KA). (A) Microfluidic device (MFD) design. The red circle indicates the top cell chamber used for confined KA stimulation. **(B)** Line graph illustrating the reactive oxygen species (ROS) production of a multi-nodal neural network following a targeted perturbation (KA stimulation). There is a clear difference between the ROS production in the target cell chamber compared to the two other chambers (5-minute intervals between datapoints). **(C)**, **(D)** and **(E)** Representative images of fluorescently labelled ROS from each of the cell chambers, from the top, middle and bottom chamber, respectively, 45 min after the perturbation. **(F)** Tiled image of the middle cell chamber of a multi-nodal neural network fluorescently labelled with Calcein-Am (green) and Ethidium homodimer-1 (red) 24 h post-KA. **(G)** Close-up of the area chosen for analysis. **(H)** Bar-graph showing the percentage of viable cells counted in each chamber, for each condition with standard deviation bars. ns ($p > 0.05$). No significant difference was found by repeated measures two-way ANOVA between chambers within the same condition ($F(1.432, 17.24) = 1.214$, $p = 0.3063$), nor by Tukey´s post hoc test between the KA and sham stimulation (PBS) within the same group for both control and LRRK2 neural networks, with

p = 0.9996 and *p* = 0.1921, *n* = *4 (2)* respectively), demonstrating that the transient perturbation was sublethal as intended. n = images (neural netwoks).

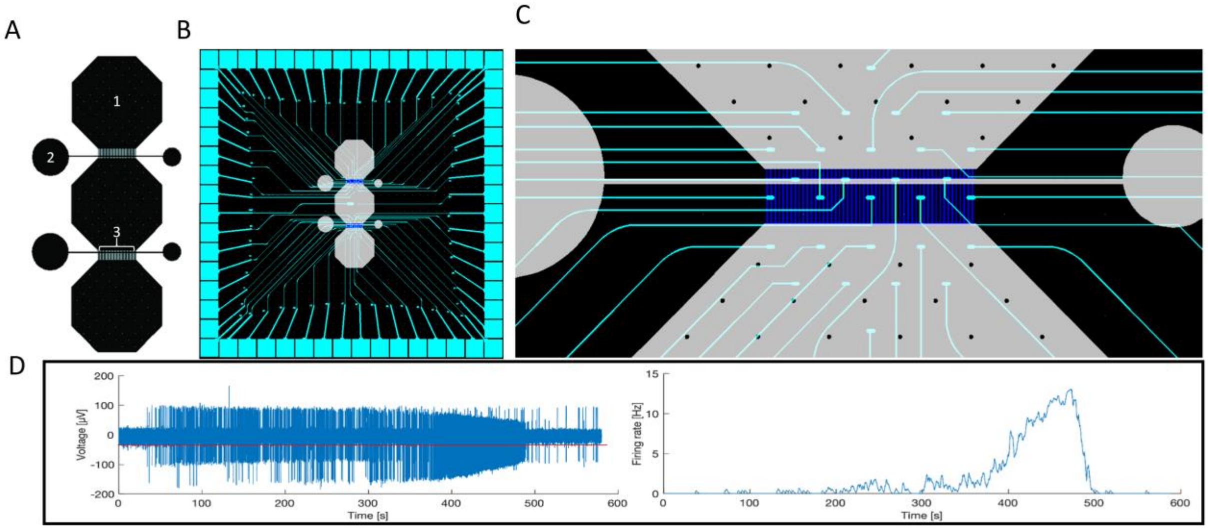

Supplementary Figure 2 Microfluidic device (MFD) interfaced with a multielectrode array. (**A)** MFD design: (1) indicates the top cell chamber, (2) the inlet/outlet MFD areas connected via the synaptic compartment, and (3) the tunnels in which neuronal axons and dendrites grow. By using different tunnel lengths, directionality of the inter-nodal connectivity can be controlled; mainly the axons from the top and bottom cell chambers connect to the dendrites and axons from the middle cell chamber in the two synaptic compartments on either side of the middle chamber. **(B)** Outline of the MFD interfaced with the custom made multielectrode array for electrophysiological investigation. **(C)** Electrode layout in the area connecting the bottom and middle cell chamber. **(D)** Shows the electrophysiological response of an LRRK2 G2019S multi-nodal neural network to the transient perturbation (KA). The graph on the left shows the activity measured at a single electrode (63) located in the top cell chamber of an LRRK2 G2019S neural network during the perturbation. The red line indicates the threshold set for spike detection. The firing rate (Hz) profile of a single neuron recorded by the same electrode is plotted on the right side of the image. A transient, drastic increase in firing rate (from 2Hz to 14Hz) can be observed 350–500 s into the recording, followed by an abrupt activity drop.

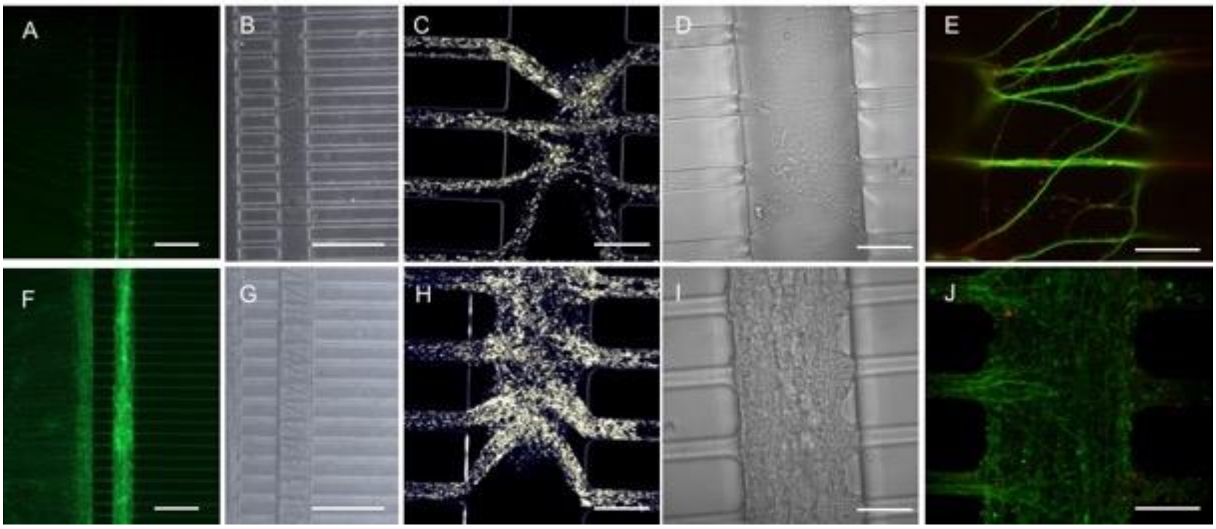

Supplementary Figure 3. Self-organization of neurons into multi-nodal networks with aberrant morphology. Microscopy images of the synaptic compartments taken during each assay demonstrate differences in neurite morphology between **(A) - (E)** isogenic control neural networks and **(F) – (**J) LRRK2 neural networks. Panels **(A)** and **(F)** show networks stained with the live stain Calcein-Am (green) (100µm scale bar), while **(B)** and **(G)** (100 µm scale bar), as well as **(D)** and **(I)** (30µm scale bar) show brightfield images. Panels **(C)** and **(H)** show live mitochondria labelled with tetramethylrhodiamine (TMRM) (30µm scale bar), while panels **(E)** and **(J)** show networks stained with the presynaptic marker Piccolo (green), the post synaptic marker PSD95 (red) (20µm scale bar).

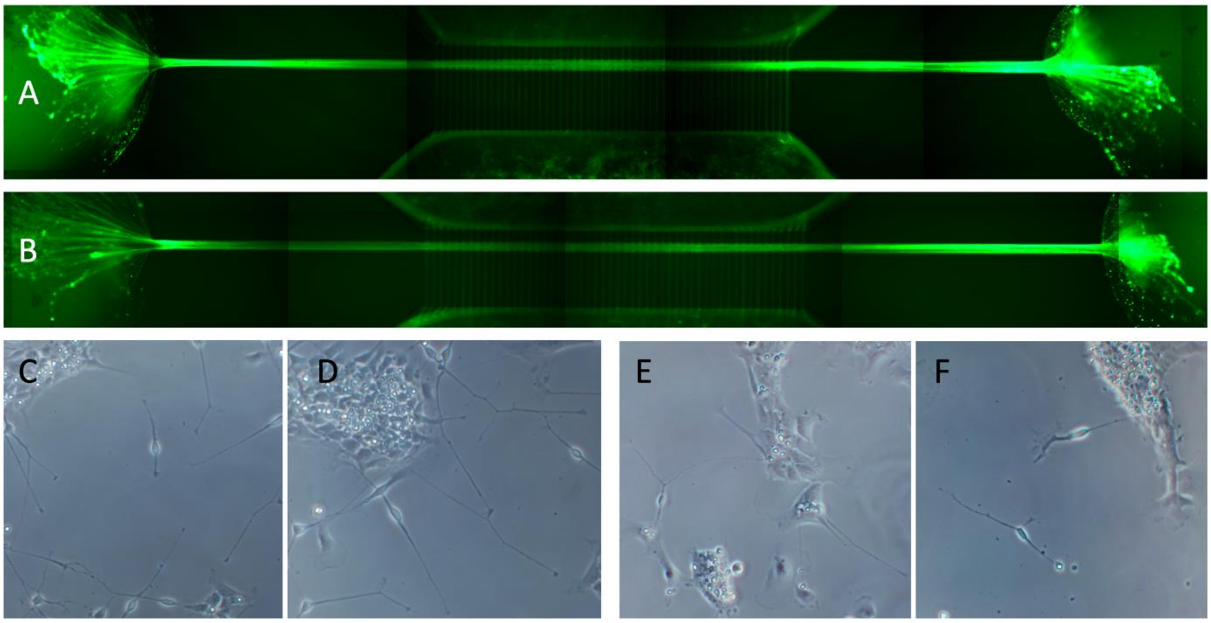

Supplementary Figure 4. Aberrant neurite outgrowth and growth cone differences in LRRK2 neural networks versus isogenic controls. (A), (B) show LRRK2 neural networks

labelled with the fluorescent Calcein-AM, where massive bundles of neurites can be seen exiting at both ends of the synaptic compartments (the inlet and outlet), where there are no cells to connect up with. These bundles are perpendicular to the axon tunnels interconnecting the cell chambers. **(C), (D)** shows brightfield images of LRRK2 NSCs, and **(E), (F)** of control neural networks after 1 day of differentiation, demonstrating differences in neurite outgrowth and growth cone profile, i.e. qualitatively more growth cones and branching neurites present in the LRRK2 neurons compared to controls.

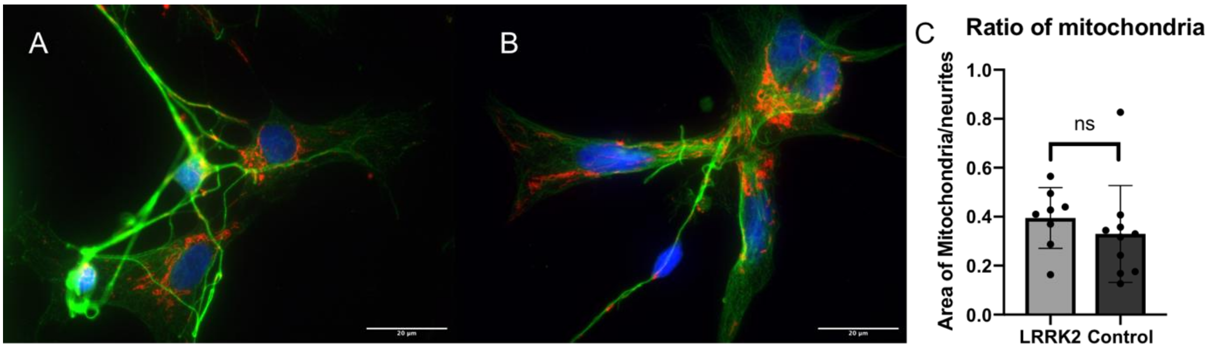

Supplementary Figure 5, Immunocytochemistry of mitochondria. (A) LRRK2 neural network with fluorescently labelled mitochondria (red (ab3298)), total-alpha synuclein (green) and hoechst counterstain (blue). **(B)** equivalent image of a fluorescently labelled control neural network (100X objective). **(C)** bar graph of the ratio calculation of mitochondria contained within samples from both the LRRK2 and isogenic control neural networks at baseline, with example images used for the calculation displayed in panel **(A)** and **(B)**. No statistically significant difference was found between the groups in mitochondrial content (ratio = area of fluorescently labelled mitochondria/ area of fluorescently labelled alpha-synuclein) (p=0.4293) by an unpaired t-test. Each dot represents a different sample (100X image) investigated, where N_{LRRK2}=8, and $N_{Control}$=10.
