# Supplementary material for: Micro-and mesoscale aspects of neurodegeneration in engineered human neural networks carrying the LRRK2 G2019S mutation
Source: Front Cell Neurosci. 2024 Apr 5;18:1366098. doi: 10.3389/fncel.2024.1366098 (PMC11026646; doi:10.3389/fncel.2024.1366098)
Supplement: Supplementary file 2 [file Table_1.docx]

**Supplementary materials and methods**

iPSC-derived H9N neural stem cells (ax0019) (control) and iPSC-derived H9N neural stem cells homozygous inserted with the LRRK2 G2019S (ax0310) purchased form Axol Bioscience were used for the experiments. Both lines are derived from the same donor dermal fibroblasts (female, 64 yr). General gene expression profiles from Axol’s iPSC-derived NSCs is published in Gene expression omnibus (GEO) of the National Center for Biotechnology information (NCBI) (<https://www.ncbi.nlm.nih.gov/geo/query/acc.cgi?acc=GSE61358> ).

Furthermore, gene editing and genotyped example data, as well as the CRISPR plasmid donor scheme for the LRRK2 line (ax0310) is shown below with the permission of Axol Bioscience.


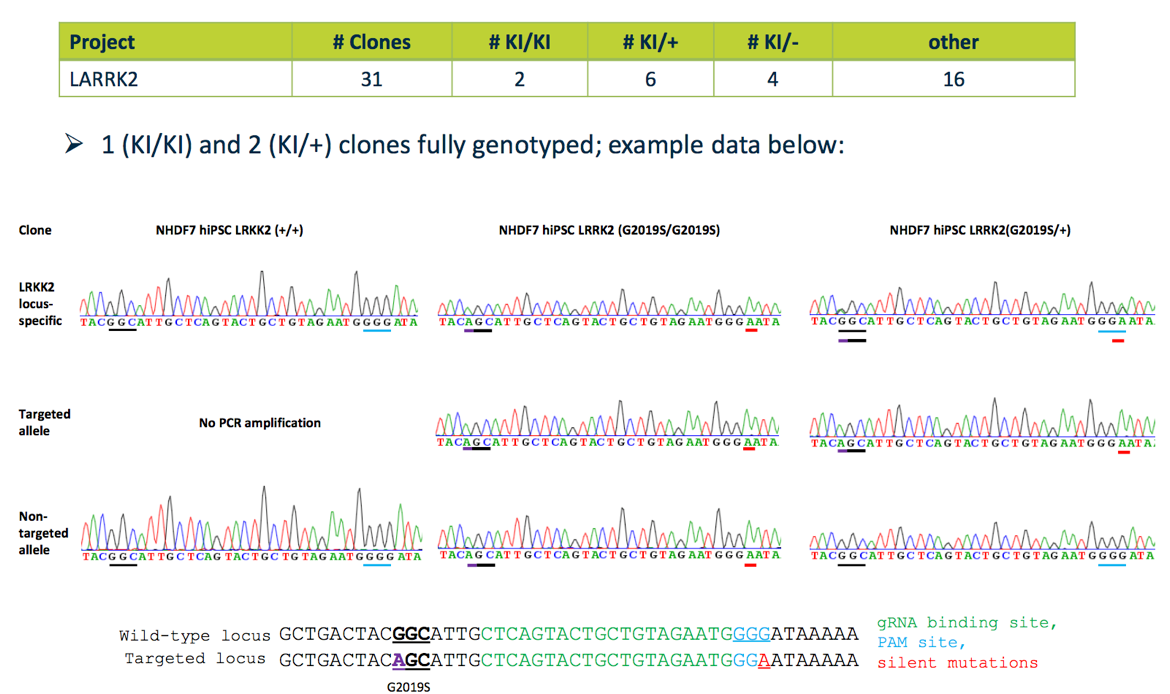


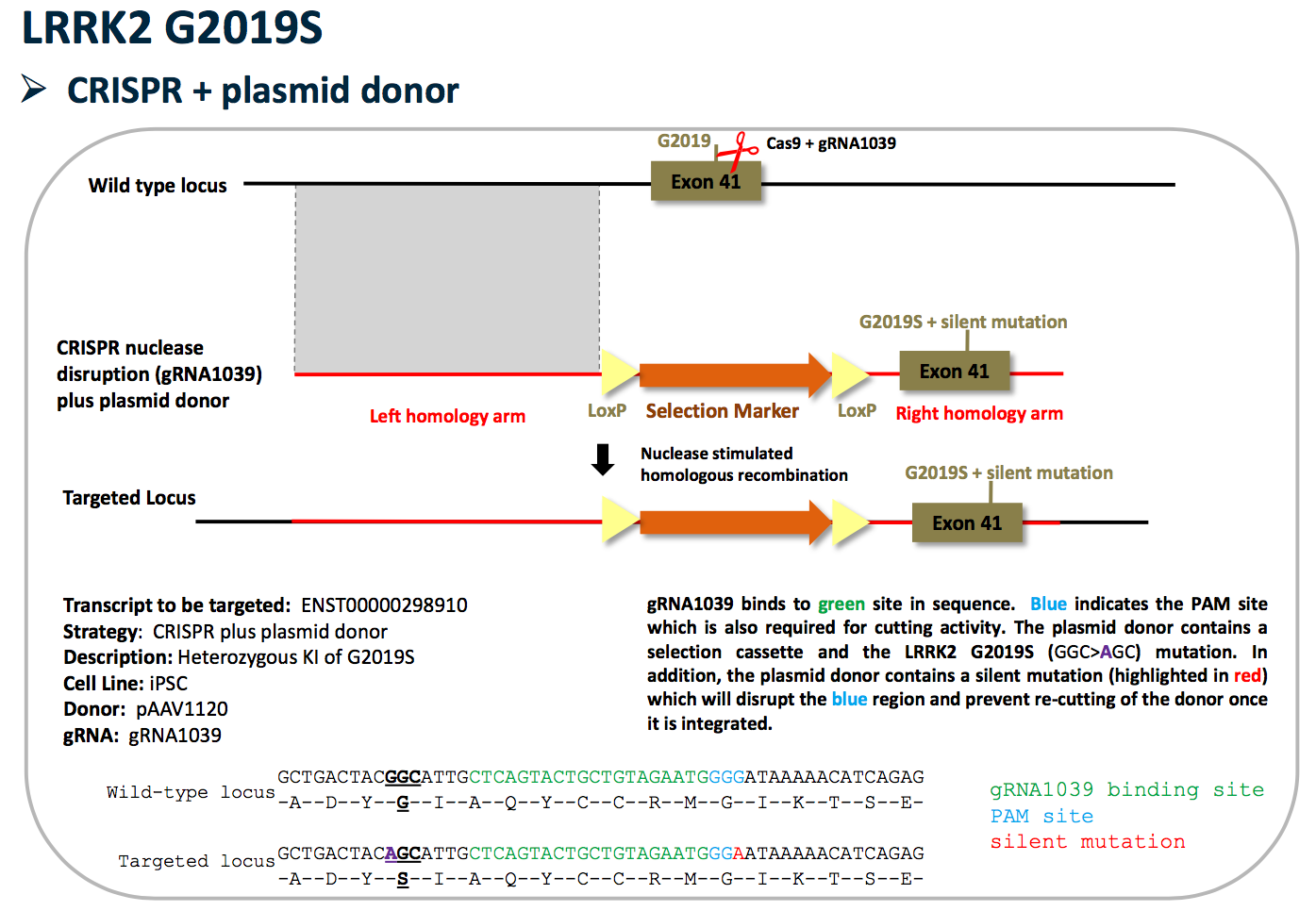


**Protocol for culture of H9N NSCs (ax0019 and ax0310), adapted from Axol Bioscience’s protocol (Human iPSC-derived Neural Stem Cell master protocol (version 5.0)**

**System A**

List of media and supplements

Neural Expansion -XF Medium (ax0030-500)

Neural Maintenance - XF Medium (ax0032-500)

Neual Differentiation – XF Medium (ax0034-125)

Sure Growth recombinant human FGF2 (ax0047)

Sure Growth X recombinant human EGF (ax0047X)

0.01% Poly-L-Ornithine solution (PLO) (Sigma, P4967)

Natural Mouse Laminin (Thermo Fisher Scientific, 23017015)

L15 Lebovitz medium (Sigma, L5521)

KnockOut serum replacement (KOSR, Thermo Fisher Scientific, 10828010)

Sodium Bicarbonate (S5761, Sigma Aldrich)

Pen-Strep (12140122, Gibco)

Rock inhibitor (T0503, Sigma Aldrich)

L15/laminin coating solution

3ml L15 Medium

48ul Natural mouse laminin (1mg/ml)

75ul Sodium Bicarbonate

Day before seeding:

Coat one well of a 6-well plate with PLO for 2 hours in incubator, wash with sterile MQ water, and leave overnight with L15-laminin in fridge.

Thaw an aliquot of Expansion medium (from -80 freezer) overnight in fridge

(Protect from light, can be kept for 1 week if supplemented (EGF, FGF2), 2 weeks if not)

Day of seed:

Prepare Neural Expansion medium with supplements (for spinning and seeding).

10ml Neural Expansion medium

2ul FGF2 (20ng/ml) (stock is 100ug/ml)

2ul EGF (20ng/ml)

10ul Rock Inhibitor (only when thawing/ splitting)

100ul Pen-Strep

Aspirate coating and add Neural Expansion medium to the well (ca 1,5ml) and return to incubator until seeding.

Thaw vial of cells in water bath (no shaking)

Pre-coat pipette with KOSR, (dropwise add cells to tube containing 10ml warm expansion medium. Spin for 200g x 5 min. Aspirate supernatant, precoat pipette with KOSR, resuspend cells in 0,5-1ml expansion medium and seed in 6-well.

Expansion:

Every 2 days, replace all the medium with Neural Expansion medium (supplemented with FGF and EGF). When the culture is 70-80% confluent, they are ready to undergo passage. This usually takes a long time (> one week). Do not expand more than 3 passages.

Splitting:

1/2 split. Coat 2 6-well wells.

Thaw a vial of Axols “Unlock” (aliquoted and stored in -80 in a box at the bottom).

Prepare Expansion medium (+ Rock inhibitor).

Aspirate media and rinse surface with DPBS--. Add Unlock (1ml /10cm^2), place in incubator for 5 min. Use 4x (unlock volume) of Neural expansion medium to stop reaction. Spin for 200g x 5min. Aspirate and resuspend in supplemented Neural Expansion medium, seed 50% in each of the 6-wells.

Synchronous Differentiation
When you have expanded long enough and are ready to start differentiating:

Full media change to Neural Expansion medium without supplements. Thaw aliquot of Neural Differentiation medium overnight in fridge.

24 hours later, conduct a full media change to Neural Differentiation medium (no supplements)

Every three days change 50% of the medium with fresh Neural Differentiation medium. A pure neuronal population takes anywhere between 3-10 days to achieve (dependent on the confluency, <60% = 3 days ++, >60% confluency takes up to 10 days, fully confluent may never become pure).

Maintenance

When fully differentiated, thaw an aliqot of Neural Maintenance medium overnight in fridge. Replace half of the medium with Neural Maintenance medium. 24 hours later, replace half the medium with Neural Maintenance.

After this, replace 50% of the medium with Neural Maintenance medium every 3 days.
Might have to add extra laminin if the cells start to detach.
